# The relationship between acculturative stress and depression among international students in China: a moderated chain mediation model

**DOI:** 10.3389/fpsyg.2026.1782600

**Published:** 2026-04-02

**Authors:** Wenyan Zhang, Qinghe Peng, Jiabao Chen, Hao Zhang

**Affiliations:** 1 College of Pharmaceutical Economics and Management, Anhui University of Chinese Medicine, Hefei, Anhui, China; 2 College of Integrated Traditional Chinese and Western Medicine, Anhui University of Chinese Medicine, Hefei, Anhui, China

**Keywords:** a moderated chain mediation model, acculturative stress, coping style, international students in China, perceived social support, psychological resilience

## Abstract

**Background:**

Depression among international students in China warrants attention. Although an increasing number of studies have highlighted depression and its potential protective factors, fewer studies have simultaneously analyzed both individual and social factors to elucidate the pathways that predict depression. Furthermore, there exists a limited body of literature on depression among international students in China, both domestically and abroad.

**Methods:**

A total of 320 international students from China completed the Acculturative Stress Scale, the Resilience Scale, the Brief Coping Style Scale, the Perceived Social Support Scale and the Depression Scale.

**Results:**

Acculturative stress was positively correlated with depression among international students. This association was jointly mediated by psychological resilience and coping styles, with the mediators operating both independently and sequentially. Moreover, perceived social support moderates the serial mediating effect of psychological resilience and coping styles between acculturative stress and depression.

**Conclusion:**

A moderated chain mediation model was developed to investigate the mediating role of resilience and coping styles and the moderating role of perceived social support. These findings provide theoretical and practical implications for preventing depression and better managing acculturative stress among international students in China.

## Introduction

Since the early 20th century, international student education in China has experienced rapid development. As an emerging regional center for studying abroad, China’s international academic programs have undergone significant expansion (Glass and Cruz, 2022). Official statistics from China’s Ministry of Education reveal that by 2018, the country hosted learners from 196 nations and territories across 1,004 universities and colleges, establishing itself as Asia’s largest host nation for overseas scholars. According to incomplete statistics, foreign learners in Chinese academic institutions were expected to surpass 360,000 by the end of 2022 (Ministry of Education of the People’s Republic of China, 2025).

As China’s international education sector expands, domestic scholars’ research on overseas learners has increased, particularly regarding their mental health challenges. Studies demonstrate that international cohorts exhibit lower mental health literacy compared to domestic peers (LaMontagne et al., 2023), potentially exacerbating their vulnerability to unaddressed psychological issues (Mori, 2000). Clinical surveys reveal also elevated rates of affective disorders among China’s international student population, including generalized anxiety, major depressive episodes, obsessive-compulsive symptoms, interpersonal sensitivity, and paranoia (Chen and Youshun, 2023). Depression emerges as the most concerning condition, demonstrating high prevalence in the population and may result in serious consequences such as disability and death (Smith, 2014). Epidemiological data show depression rates exceeding 50% among this demographic (Gu et al., 2020), surpassing the national average (The State Council Information Office the Peoples Republic of China, 2021). This crisis peaked during the 2019 COVID-19 pandemic surge, with regional studies documenting depressive symptoms reaching 59.4% (Wang et al., 2020).

In studies on depression, life events and ongoing stressors in life are important factors leading to depression (Hammen, 2015; Mazure, 1998). Due to the convergence of educational, sociocultural adjustments, and economic pressures, psychological distress is highly prevalent among college students (Gilbert et al., 2021). As overseas students adjust to new cultural environments, they often suffer from the “culture shock” phenomenon (Oberg, 1960), acculturative stress is prominent stressor, closely linked with depression (Liu et al., 2021; Minutillo et al., 2020) and represents a critical determinant of psychological distress (Cao et al., 2021). Beyond acculturative stress, empirical analyses have identified multiple intersecting variables—including demographic markers (gender, age, ethnicity), academic tenure duration, linguistic proficiency, and crucially, perceived social support—that collectively modulate depression risk., and the results show that social support is the most potent protective factor against severe depressive episodes in this population (Sümer et al., 2008). In addition, multiple studies have identified resilience (Singh, 2020) and coping styles (Akhtar and Kroener-Herwig, 2017) as important factors for international students’ health outcomes.

Empirical investigations reveal that international students face problems such as difficulties in adapting to Chinese culture (Wu et al., 2021). Research indicates that the level of acculturative stress among international students in China is higher than that among their counterparts in developed countries. Three factors associated with this higher level of acculturative stress are being unprepared for studying abroad, being married, and holding religious beliefs (Yu et al., 2014). A review of factors influencing the life satisfaction and adaptability of international students in China reveals elements similar to those found in other national contexts, such as acculturation issues, personal coping ability, financial stressors, and perceived discrimination. However, these students in China also face unique challenges, primarily manifested in language barriers, the limited availability of campus support services and social integration with Chinese students (Jiang et al., 2020). When it is difficult to accept and identify with Chinese culture in the early stages, integration into the local academic social life in China becomes challenging, which may result in adverse psychological reactions (Li et al., 2019) In the Chinese cultural context, Chinese college students with high psychological resilience are better able to coordinate all resources to adapt to stress (Liu et al., 2015).

While global research extensively examines factors contributing to depression among international students in Western contexts, analogous studies targeting those in China remain scarce. current literature predominantly adopts a fragmented approach, treating internal psychological variables and external psychosocial variables as isolated domains rather than investigating their interconnected dynamics. Crucially, few investigations holistically assess how the interplay of acculturative stress, adaptive coping mechanisms, psychological resilience, and perceived social support collectively predicts expressive symptom development. This study bridges these critical gaps by integrating multifactorial theories to analyze depression determinants, thereby offering both conceptual advancements and actionable insights for supporting international students in China.

### Theoretical basis

Based on acculturative stress framework, acculturative stress constitutes a distinct psychological response arising from cross-cultural adaptation (Berry, 1997), and represents a psychological coping mechanism produced by individuals (Gilbert et al., 2021), manifesting as individuals’ attempts to reconcile symbolic meanings within unfamiliar sociocultural ecosystems. This theory posits a possible relationship between stress intensity and depression. Individuals mainly experience culture shock or acculturative stress when cultural conflicts are within their control; if there are significant difficulties beyond the individual’s coping capacity, serious psychological disorders may arise (Berry, 1988). When the stress load reaches a debilitating level, individuals may exhibit symptoms of anxiety or depression (Li et al., 2019).

The American Psychological Association’s perspective defines resilience as “the process of adaptation in the face of adversity, trauma, tragedy, threat, or significant sources of stress” (American Psychological Association, 2025). Based on resilience process theory, the concept of resilience has shifted from a stable characteristic to a dynamic process (Fullerton et al., 2021), which is the result of successful adaptation to stressors (Ungar, 2014). At the same time, psychological resilience helps individuals maintain stronger mental wellbeing than would normally be predicted after experiencing hardship within their specific cultural environment based on acculturative stress framework (Troy et al., 2025).

Life events, stress, coping, and illness are often studied as part of a process theory that predicts mental health outcomes (Warheit, 1979)..Coping styles encompass behavioral patterns activated during novel or unfamiliar circumstances (Beutler et al., 2010). Coping styles are closely linked to acculturative stress. Stress manifestation correlates with the amount, frequency, intensity, and duration of available coping resources (Gilbert et al., 2021). Individual coping styles are significant in dealing with acculturative stress (Carver et al., 1989) and can predict acculturative stress (Crockett et al., 2007). Research has demonstrated that coping styles can explain differences in acculturative stress, and different coping styles tend to predict different levels of acculturative stress (Akhtar and Kröner-Herwig, 2015).

Perceived social support functions as a cognitive schema wherein individuals’ established frameworks of relational quality shape the appraisal and recollection of social experiences (Lakey and Cassady, 1990). Perceived social support affects resilience and is thus associated with depression. Based on resilience process theory, resilience constitutes a multidimensional adaptive capacity shaped by both intrinsic traits and extrinsic resource interactions, such as socioeconomic status, and institutional networks (Gomez and Gudiño, 2022). Moreover, the regulatory capacity for resilience is not sustainable unless both social and physical systems come together to support adaptive behavior (Oshri et al., 2018). Supportive social networks amplify resilience through self-efficacy fortification and neurophysiological regulation, including parasympathetic nervous system engagement (Sippel et al., 2015).

Based on these theoretical perspectives, we propose that the integration of acculturative stress, resilience, coping styles, and perceived social support provides a comprehensive framework for understanding depression in this population.

### The current study

Psychometric analyses confirm a positive correlation between acculturative stress intensity and depression risk among international populations (Elif et al., 2024). Meanwhile, meta-analytic evidence further identifies depression as the predominant negative psychological sequela of prolonged acculturative strain across overseas student cohorts (Soufi Amlashi et al., 2024).

Resilience demonstrates inverse correlations with depressive disorders across clinical and non-clinical populations. Multiple resilience factors not only reduce an individual’s lifetime depression susceptibility (Lavretsky and Irwin, 2007), but also function as protective mediators of post-traumatic exposure, which significantly reduces the severity of depressive symptoms (Wingo et al., 2010). In patients with major depression, resilience reduces the risk of onset or relapse and promotes recovery (Laird et al., 2019). Previous research has shown resilience to be a mediator with significantly associated with mental health (Zhou et al., 2022). Cross-culturally, resilience operates as a critical mediator between acculturation strategies and mental health outcomes, modulating how cultural adaptation approaches predicts psychopathological risks (Wu et al., 2018). It has also been validated in related research that Latinos use cultural strengths like resilience to buffer the association between acculturative stress and somatic symptoms (Cariello et al., 2020).

How stressors are perceived and coped with may largely determine depressive responses (Olff, 1999). A survey of college students from other countries postulated that effective coping diminishes stress load and mitigates depressive symptoms (Kumar, 2016). However, research on Latino adolescents paradoxically showed amplified acculturative stress-depression linkages among those employing active coping strategies (Gomez and Gudiño, 2022). Furthermore, coping style is a key mediating variable between stressful events and related health consequences (Folkman, 1984). The acculturative stress strategies model proposes coping strategies as a key variable in the study of psycho-cultural adaptation, and it can serve as a mediating variable when coping strategies link stressors to stress responses (Li et al., 2019). Recent studies have additionally validated the mediating role of coping styles in mental health (Pereira-Morales et al., 2018).

Resilience and coping styles exhibit significant associations. High resilience may help international students adopt more appropriate coping styles and improve their ability to manage acculturative stress. Resilience is the self-regulatory capacity to maintain stable psychological functioning during crises or restore baseline functioning post-disruption (Windle, 2011). It serves as a dynamic process that also determines the ability to cope successfully (Wackerhagen et al., 2023). In a cross-cultural context, resilience is a critical enhancer of acculturative stress tolerance (Rudmin, 2009). For an international student in China, robust resilience sustains psychosocial stability through transitional phases (Bonanno et al., 2011) Leveraging proactive coping mechanisms to navigate intercultural challenges and assimilate into host environments. Resilience and coping styles can then work together to potentially predicts levels of depression. In psychopathology, coping styles in response to stress share a neurobiological basis with the identification of resilience, and psychopathological manifestations like depression (Cabib and Puglisi-Allegra, 2012). According to the Resilience Process Theory (Stainton et al., 2018), personal attributes, and the way people assess and respond to stressors (Warheit, 1979), coping styles, interact with each other, and thus may predicts depression levels. It has been shown that a chain mediation between resilience and coping styles can be formed to help students adapt to the new school environment (Wu et al., 2024) and avoid adverse psychological consequences.

Among overseas scholars, resilient dispositions demonstrate significant covariation with relational quality and perceived support within familial and academic spheres (Caqueo-Urízar et al., 2021). Resilience and social support are both positive factors and are negatively associated with depressive symptoms (Brailovskaia et al., 2017). Perceived social support creates a connection with resilience and prevents students from developing depressive (Dong et al., 2024) Moods as confirmed in related studies. Beyond resilience, perceived social support has unique implications on depression that occurs during acculturative stress. Acculturation occurs during cross-cultural adjustment when members from different cultures interact with each other through interpersonal relationships and mediating channels (Berry, 2006). Positive acculturation occurs when an individual exhibits behaviors associated with social or interpersonal competence (Luthar and Cicchetti, 2000), but depression may occur when there is a “loss” of interpersonal relationships, which may include bereavement, separation, closure, or the threat of separation (Paykel, 2001). Previous research elucidated links between perceived support, resilience, acculturative stress, and depression, therefore positioning social support as a potential moderator within resilience-mediated pathways.

Based on the reviewed literature, we propose the following hypotheses:

*H1:* Acculturative stress is associated with depressive symptoms among international students in China.

*H2:* Resilience mediates acculturative stress and depression among international students in China.

*H3:* Coping styles mediate the relationship between acculturative stress and depression among international students in China.

*H4:* Resilience and coping styles function as sequential mediators in the pathway linking acculturative stress to depression among international students in China.

*H5:* Perceived social support moderates the association between acculturative stress and resilience.

**FIGURE 1:**
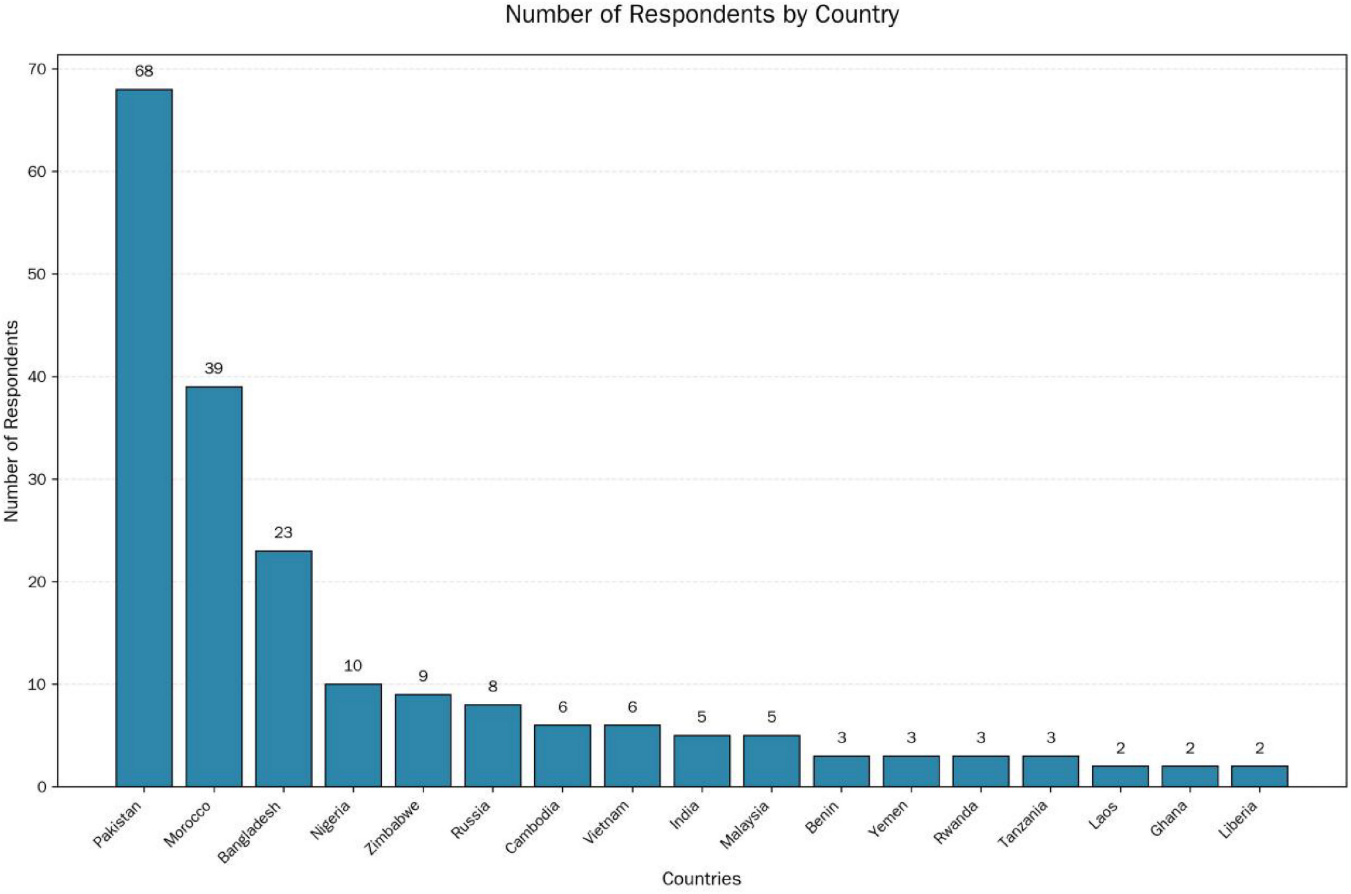
Participants regional backgrounds (based on available data). A total of 314 valid questionnaires were collected in this study. Regarding the item on nationality/country of origin, this question was not mandatory; consequently, 197 participants (62.7% of the total sample) provided information on their country or region of origin, while 117 participants left this item unanswered.

These propositions inform a moderated chain mediation model, as illustrated in Figure 1.

## Materials and methods

### Participants

**FIGURE 2:**
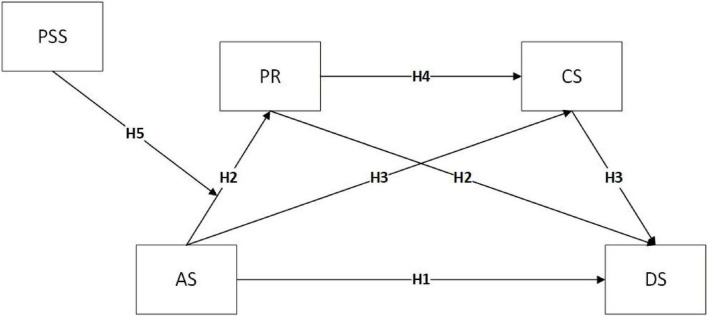
Moderated chain mediation model. Theoretical path model. AS, Acculturative Stress; PR, Psychological Resilience; CS, Coping Style; PSS, Perceived social support; DS, Depression.

International students from five representative universities of medicine, science and technology, teacher training, agriculture and forestry, and finance and economics from East China were selected as the research subjects. From 320 initial submissions, 314 valid responses were retained after eliminating non-compliant entries containing incomplete fields, contradictory answers, or repetitive response patterns, and the validity rate of the questionnaires was 98.125%. The study subjects were above 16 years of age, of whom 155 (49.2%) were males and 159 (50.8%) were females. There were 201 (64.0%) undergraduate students, 67 (21.4%) graduate students, and 46 (14.6%) post-graduate students. As shown in Figure 2, participants were predominantly from Asian and African countries, with a high concentration of individuals from Pakistan.

### Procedures

After obtaining informed consent from both the teachers and international students from the five universities, researchers administered digital questionnaires via WeChat’s mini-program interface. A randomized sampling protocol ensured voluntary participation and response authenticity. All questionnaires were filled out anonymously, and the researcher was present throughout the process to promptly address any questions. Ethical compliance was verified through formal approval by Anhui University of Traditional Chinese Medicine’s Research Ethics Committee, which authorized all recruitment and data acquisition procedures.

## Measures

### Acculturative stress

The Acculturative Stress Scale for International Students (ASSIS), pioneered by Sandhu and Asrabadi (1994) Quantifies self-reported acculturative stress experiences. It consists of 36 items with seven subscales, administered via a 5-point Likert index. Higher scores correspond to greater acculturative stress. In this study, with the full scale demonstrating excellent internal consistency (α = 0.96), subscale reliability coefficients ranged from α = 0.75 to 0.89.

### Psychological resilience

Psychological resilience was assessed using the Connor-Davidson Resilience Scale (CD-RISC) (Connor and Davidson, 2003), a 25-item scale validated across multiple studies for its psychometric robustness. Comprising five subdimensions and employing a 5-point Likert system, with higher composite scores reflecting an enhanced adaptive capacity. In this study, the scale exhibited strong reliability (α = 0.95), with subscale coefficients spanning α = 0.61–0.92.

### Coping style

Charles S. Carver developed the Brief Cope Scale (BCS) to provide researchers with a quick way to assess potentially important coping responses (Carver, 1997). It is 28 items categorized into three strategy typologies, scored on a 4-point spectrum. Differential scoring profiles were used to classify participants’ coping styles. In this study, with the full scale showing high consistency (α = 0.911) and subscales ranging from α = 0.76–0.83.

### Perceived social support

The Perceived Social Support Scale (PSSS) was developed by Zimet et al. (1988) to evaluate subjective assessments of relational support networks. This 12-item tool, structured across three domains using a 5-point Likert framework, associates elevated scores with stronger perceived support. In this study, the internal consistency was α = 0.91.

### Depression

The Depression Scale is the depression section of the Depression Anxiety and Stress Scale (DASS-21), which is based on the tripartite model proposed by Clark and Watson (Clark and Watson, 1991). The 7-item module, employing a 4-point severity scale, links higher scores to increased depressive severity. In this study, the internal consistency was α = 0.89.

### Data analysis

Quantitative analyses were executed using SPSS 26.0. A common method bias test was first conducted. Subsequent analytical phases encompassed: (1) descriptive profiling of variable distributions; (2) bivariate correlation assessments via Pearson’s coefficients; (3) predictive modeling through multivariate linear regression. To examine hypothesized mediation pathways, PROCESS 3.3 macro facilitated bootstrap analysis (5,000 iterations) for evaluating chain mediation effects of resilience and coping style between acculturative stress and depression. Finally, the moderating role of perceived social support was tested.

## Results

### Control and verification of common method variance

Given the self-reported nature of the data collection protocol, potential common method variance was evaluated using Harman’s single-factor diagnostic approach. An unrotated principal component analysis incorporating all measurement items yielded 19 components with eigenvalues > 1. The initial component accounted for 19.817% of the total variance, remaining substantially below the 40% critical threshold. These findings suggest minimal influence of methodological bias on the study’s outcomes.

### Descriptive statistics and correlation analysis

Descriptive statistics and intervariable correlations are presented in Table 1. The correlation analysis revealed significant associations between acculturative stress and all measured constructs, including a negative correlation with resilience and perceived social support and a positive correlation with coping styles and depression. Resilience exhibited positive covariation with coping styles, perceived social support, while inversely relating to depression; coping style showed no significant linkage to perceived social support but correlated positively with depression. Perceived social support was positively correlated with depression. The proposed model demonstrated a good fit to the data (Table 2), supporting the hypothesized relationships. All predictor variables (acculturative stress, resilience, coping styles) demonstrated statistically significant predictive capacity for depression (*p* < 0.05*). Furthermore, there was no autocorrelation and multicollinearity between acculturative stress, resilience, coping style, and perceived social support.

**TABLE 1:** Means, standard deviations, and correlations for study variables.

Variable	M±SD	AS	PR	CS	DS	PSS
l. AS	90.20 + 23.92	−	−	−	−	−
2. PR	94.16 + 19.27	−0.218**				
3. CS	65.19 + 15.19	0.177**	0.151**			
4. DS	11.80 ± 4.88	0.433**	−0.268**	0.355**		
5. PSS	45.09 ± 9.37	−0.304**	0.425**	0.022	−0.184**	

*M*, mean; *SD*, standard deviation. Correlations are based on pairwise *N* = 314.

**p < 0.01.

**TABLE 2:** Analysis of linear regression results (*N* = 314).

	Unstandardized coefficient		Standardized coefficient	*t*	*P*	Covariance diagnose	
Variable	β	Standard error	Beta			VIF	Tolerances
(Constant)	4.565	1.974	−	2.312	0.000	−	−
AS	0.066	0.010	0.322	6.381	0.021	1.167	0.857
PR	−0.064	0.013	−0.254	−4.810	0.000	1.276	0.784
CS	0.107	0.016	0.336	6.921	0.000	1.074	0.931
PSS	0.008	0.028	0.015	0.278	0.781	1.295	0.772
*R* ^2^			0.324				
Adjustment *R*^2^			0.315				
F			*F*(314) = 37.003, *p* = 0.000				
D-W value			1.959				

### The chain mediating effect test

The hypothesized mediation pathways were examined using PROCESS 3.3 (Model 6), combined with the Bootstrap method to obtain the effect values, standard errors, and Bootstrap test results for different paths. The chain mediation model was established with acculturative stress as the independent variable (X), depression as the outcome (Y), and resilience (M1) and coping style (M2) as the mediator variables; Table 3 illustrates the specific test results.

**TABLE 3:** The chain mediating effect test (*N* = 314).

Predictive variable	Outcome variable: PR				Outcome variable: CS				Outcome variable: DS			
	β	SE	95% CI		β	SE	95% CI		β	SE	95% *CI*	
AS	−0.176***	0.045	−0.264	−0.089	0.139***	0.036	0.069	0.21	0.065***	0.01	0.046	0.085
PR	0.048				0.155**	0.044	0.067	0.242	−0.063***	0.012	−0.088	−0.039
CS					0.068				0.107***	0.016	0.076	0.137
*R* ^2^									0.322			
F	15.659***				11.270***				48.929***			

**p < 0.01.

***p < 0.001.

Analytical outcomes (Table 3) demonstrate that acculturative stress significantly positively predicted depression (β = 0.065, *p* < 0.001) and coping styles (β = 0.139, *p* < 0.001), negatively predicted resilience (β = −0.176, *p* < 0.001); resilience significantly positively predicted coping styles (β = 0.155, *p* < 0.01) and negatively predicted depression (β = −0.063, *p* < 0.001). Coping style significantly positively predicted depression (β = 0.107, *p* < 0.001). The mediation effect showed that resilience and coping style functioned as significant mediators, with a knock-on mediating effect between acculturative stress and depression in Table 4. With a total indirect effect size of 0.023, the chain mediation model accounted for 26.16% of the total effect.

**TABLE 4:** Bootstrap analysis of mediation effects.

Categories	Effect	Boot SE	*95% CI*		Ratio of indirect to total effect	The ratio of indirect to direct effect
Total effect	0.088	0.010	0.000	0.068	−	−
Direct effect	0.065	0.010	0.000	0.046	−	−
Total indirect effect	0.023	0.006	0.012	0.036	26.16%	35.43%
AS→ PR→ DS	0.011	0.004	0.004	0.021	12.57%	17.02%
AS→ CS→ DS	0.015	0.005	0.005	0.027	16.87%	22.85%
AS→ PR→ CS→ DS	−0.003	0.002	−0.0066	−0.0006	3.41%	4.62%

### Moderating effect test

To investigate the hypothesized moderation effect, Model 83 within the PROCESS 3.3 macro was implemented. Table 5 shows that perceived social support emerged as a robust negatively predictor of resilience (β = 0.699, *p* < 0.001). Additionally, acculturative stress and its interaction with perceived social support significantly predicted resilience (β = −0.017, *p* < 0.001). Therefore, these findings confirm that perceived social support significantly moderated the relationship between acculturative stress and resilience.

**TABLE 5:** Moderated chain mediating effect test (*N* = 314).

Predictive variable	Outcome variable: PR				Outcome variable: CS				Outcome variable: DS			
	β	SE	95% CI		β	SE	95% CI		β	SE	95% CI	
AS	−0.083*	0.042	−0.166	−0.0002	0.141***	0.036	0.071	0.212	0.065***	0.010	0.046	0.085
PSS	0.699***	0.111	0.479	0.917	0.158**	0.044	0.070	0.245	−0.063***	0.012	−0.088	−0.039
PR	−0.017***	0.004	−0.025	−0.009								
AS*PSS					0.069				0.107***	0.016	0.077	0.138
CS	0.023											
*R* ^2^									0.322			
F	30.983***				11.543***				48.459***			

*p < 0.05,

**p < 0.01,

***p < 0.001.

### Model of moderated chain mediating effect test

**FIGURE 3:**
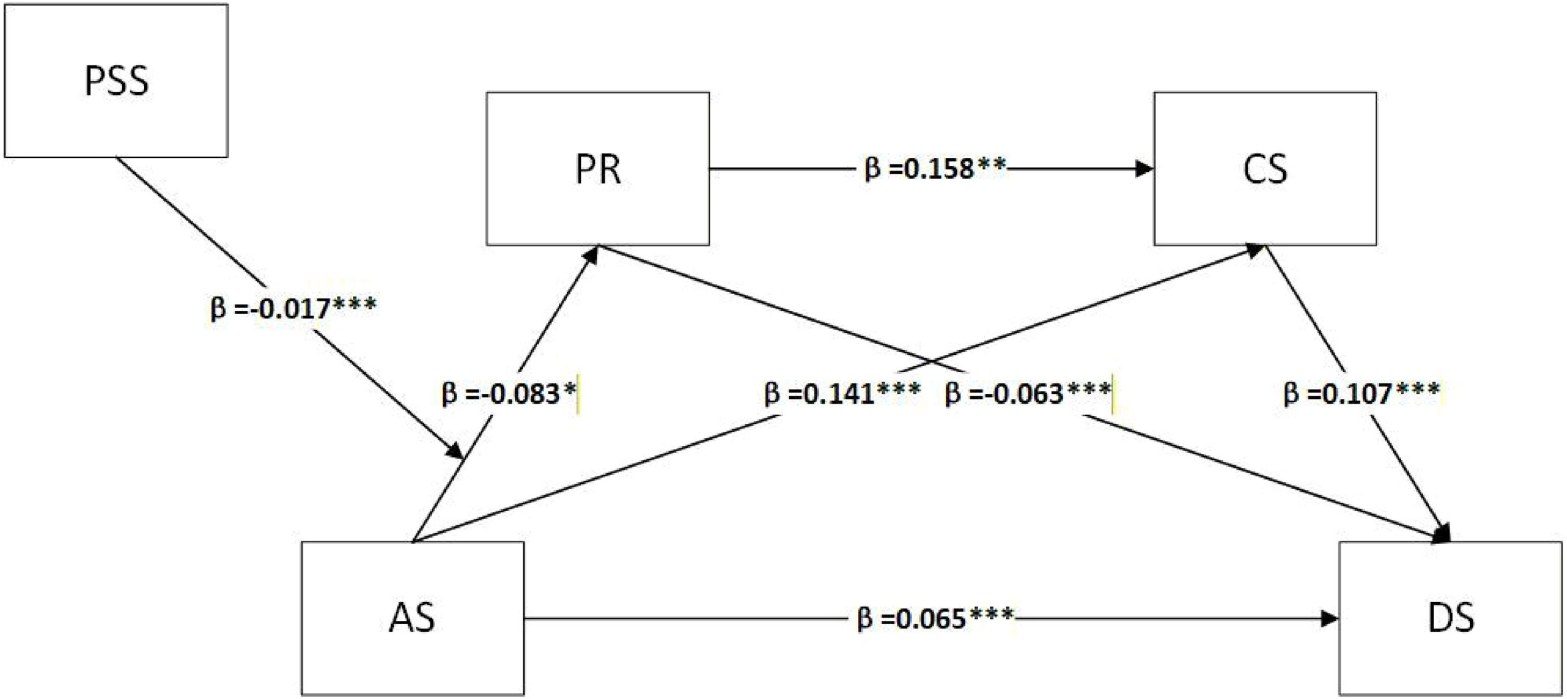
Moderated chain mediator model. *p < 0.05, **p < 0.01, ***p < 0.001.

Figure 3 demonstrates that the interaction between acculturative stress and perceived social support has a significant association on resilience (β = −0.017, *p* < 0.001). This indicates that perceived social support significantly moderates the relationship between acculturative stress and resilience. The analysis of moderated chain-mediated effects was conducted using analysis of variance, with the significance of these effects assessed by directly evaluating the difference in mediated effects (Porter et al., 1994), to synchronously test the moderating role of perceived social support between acculturative stress and resilience in the chained mediation model. As outlined in Table 6, under conditions of diminished perceived social support (M − 1 SD), the chain-mediated pathway “acculturative stress→ resilience→ coping styles→ depression” is not significant. Conversely, heightened perceived social support (M + 1 SD) revealed statistically robust mediation. At the same time, comparative analysis showed substantial differences in the effect values of which indicated that perceived social support could significantly moderated the indirect effects of relationship between acculturative stress and depression via resilience and coping styles. The final model yielded a significant index of moderated mediation, supporting the conditional indirect effect [Index = −0.0003, 95% CI (−0.006, −0.0001)].

**TABLE 6:** Model of moderated chain mediating effect test (*n* = 314).

Moderator variable	Effect	AS→PR→CS→DS	Index of moderated mediation	
		95% CI	Index	95% CI
Low-level PSS	0.0011	(−0.0017, 0.0040)	−0.0003	(−0.006, −0.0001)
High-level PSS	−0.0042	(−0.0087, −0.001)		
different (high-low)	−0.0053	(−0.0114, −0.0010)		

## Discussion

### The relationship between acculturative stress and depression

This investigation confirmed a significant positive association between acculturative stress and depression, supporting hypothesis 1. Despite the differences in cultural environments across countries, international students in China, similar to other international student populations (Driscoll and Torres, 2021), generally face acculturative challenges, reinforcing stress exposure as a robust predictor of depressive outcomes. When international students in China are separated from their original cultural environments, they significantly perceive discrepancies from their culture and host country (especially Western-origin students adapting to Chinese societal structures). Since cultural changes in life (either positive or negative) induce stress (Berry, 2006), the emergence of acculturative stress is inevitable. However, the manifestation of depressive symptoms remains non-universal among stress-exposed students, highlighting pronounced individual variations in stress perception and mental health outcomes. Research underscores the necessity of prolonged adaptation periods for full sociocultural integration (Li et al., 2019). In the process of adapting to Chinese culture, if acculturative stress is perceived as a major challenge and the stress load exceeds the individual’s tolerance, additional psychological resources are required, significantly increasing the probability of depressive symptoms. Therefore, to prevent depression and other psychological problems among international students, it is necessary to focus on the typical difficulties they encounter in Chinese culture and establish targeted support mechanisms.

### The mediating role of psychological resilience

This research substantiates resilience as a pivotal mediator in the acculturative stress-depression nexus among international students in China, which confirms that this group can buffer the acculturative stress through resilience and thus supports the second hypothesis. This not only tests previous research on resilience, which shows that high resilience can help people handle stress better and is conducive to maintaining health (Elif et al., 2024). The cross-cultural applicability of resilience metrics facilitates extending resilience frameworks from individual coping mechanisms to broader public health paradigms (Denckla et al., 2020). While international scholars in China encounter comparable acculturative pressures, depressive outcomes remain non-uniform across the population. This variability underscores resilience’s explanatory power in differential stress responses, transcending mere exposure intensity (Mc Gee et al., 2018). Students exhibiting heightened resilience demonstrate greater success in achieving sociocultural integration, thereby attenuating acculturative stress and diminishing depression risk. Consequently, resilience operates as a critical safeguard, enabling international learners to navigate cultural transitions while preserving mental health.

### Mediating role of coping style

This study revealed that coping style serves as a mediator between acculturative stress and depressive outcomes among international students in China, thereby validating Hypothesis 3. The findings indicate that the assessment of acculturative stress and coping habits by international students together determine whether it leads to depression. The combination of risk factors and cultural contextual stressors for international students in China may lead to different coping styles, which are differentially linked to psychological distress (Cheng et al., 2023). Acculturative stressors can be categorized into “controllable and uncontrollable stressors,” and if they reduce emotional responses to uncontrollable factors and focus on action-oriented responses to controllable factors, it can help them better adapt to the Chinese environment. Conversely, maladaptive coping patterns-such as avoidance or rumination-correlate with impaired cultural adaptation and heightened vulnerability to depression. Therefore, to avoid depression, international students should adopt appropriate coping styles to deal with acculturative stress.

### Mediating roles of resilience and coping style

The present study additionally found that resilience and coping styles had a chain mediating role between acculturative stress and depression among international students in China, and the results corroborated hypothesis 4. This further demonstrates that resilience and coping styles as adaptive to stressors and adversity (DiCorcia and Tronick, 2011) are two interrelated types of individual differences, and a combination of both has the potential to ameliorate or exacerbate the adverse responses of these experiences (Chen, 2016). The resilience and coping styles of international students in China, when facing acculturative stress, may interact to predict their level of depression. High resilience is important in promoting positive coping in a new school environment (Bonanno et al., 2011). Within novel academic circumstances, enhancing resilience not only helps them maintain a healthy psychosocial state, but also implies utilizing internal and external resources to help them cope with difficulties (Alvord and Grados, 2005), which in turn alleviates maladaptive psychiatric disorders such as depression. On the contrary, diminished resilience correlates with maladaptive coping approaches, amplifying the likelihood of acculturative stress precipitating depression. Therefore, institutional interventions should prioritize enhancing resilience and promoting adaptive coping techniques to disrupt this pathway and safeguard students’ mental health.

### The moderating role of perceived social support

This research revealed that perceived social support negatively moderated the serial mediation model, supporting Hypothesis 5. Compare with prior research (Dong et al., 2024), heightened perceived social support paradoxically amplified the adverse association of acculturative stress on psychological resilience. This divergence may stem from contextual cultural dynamics influencing support efficacy. Under normal circumstances, individuals who meet difficulties they cannot handle initially rely on familial networks for assistance. If these resources are not available, they turn to friends whom they can confide in and who can help (Gilbert et al., 2021). Such support systems typically function as safeguards for resilience, with the combination of emotional and material resources they provide considered one of the social factors influencing resilience (Wilks and Croom, 2008). Robust social support networks are widely recognized for fostering resilience and cultivating positive psychological outcomes (Costa et al., 2017). In contrast, the relatively introverted dating style in China leads to these students in China having fewer friends to confide in, and the social support they feel in China may not be high-level or positive, which may not effectively promote the development of resilience in international students in China.

### Implications

This study has theoretical significance. It utilizes theories of acculturative stress, cognitive appraisal of stress, resilience, and others to integrate multiple dimensions of cultural, psychological, social, and personal factors into a quantifiable model. This study explores not only the protective defense mechanisms that shape resilience and coping styles counteracting depressive outcomes during cultural transitions but also the role of perceived social support as a moderator between acculturative stress and resilience, which takes full advantage of the characteristics of resilience, understanding it as an ecological process-one where resilience emerges through synergistic interactions across biological, psychological, and sociocultural systems. This systemic perspective demonstrates how individuals dynamically mobilize internal and external resources when confronting cultural stressors, enabling psychological recalibration rather than mere risk avoidance (Ungar and Theron, 2020).

Furthermore, this study is of practical significance. The increasing movement of international students, particularly within China’s rapidly expanding academic landscape, has amplified concerns regarding psychological vulnerabilities, as this population demonstrates heightened susceptibility to depressive disorders compared to domestic peers (Smith, 2014). While acculturative stress remains an inescapable hurdle during cultural adaptation, its progression to clinical depression serves as a critical indicator of students’ capacity to assimilate into China’s sociocultural milieu. Although acculturative stress is significantly positively correlated with depression, there are still groups of international students with good psychological status. Therefore, we need to pay more attention to these evidence-based strategies, enabling targeted interventions in the future, such as reducing the level of acculturative stress, enhancing resilience, adopting positive coping styles, and increasing social support for international students.

These findings offer several practical implications for university support services. First, the significant mediating role of resilience suggests that student counseling services could develop targeted resilience training programs to help international students buffer the negative effects of acculturative stress. Second, given the buffering effect of perceived social support, universities could leverage international affairs offices to facilitate mentorship programs and create structured social support opportunities, such as peer connection events or cultural integration workshops, thereby mitigating the risk of depression.

### Limitations

The investigation’s methodological constraints include three primary areas for refinement. Geographically, the sampling framework was restricted to Eastern China, overlooking regional sociocultural variations that may predict acculturative adaptation outcomes. Expanding recruitment to encompass broader provincial representation would enhance ecological validity.

Additionally, the modest sample size, attributable to East China’s limited international student population and potential recruitment barriers. Self-selection biases may further skew findings, as psychologically distressed students might avoid participation.

Future research should adopt cross-regional comparative designs to elucidate location-specific cultural dynamics while integrating longitudinal analyses tracking mental health trajectories. Diversifying cultural cohorts and incorporating moderators like pre-migration preparedness, digital acculturation patterns, and institutional support structures would yield richer insights into China’s international student mental health landscape.

## Data Availability

The raw data supporting the conclusions of this article will be made available by the authors, without undue reservation.
